# Immunoregulatory Role of HLA-G in Allergic Diseases

**DOI:** 10.1155/2016/6865758

**Published:** 2016-06-20

**Authors:** Giuseppe Murdaca, Paola Contini, Simone Negrini, Giorgio Ciprandi, Francesco Puppo

**Affiliations:** ^1^Department of Internal Medicine, Scleroderma Unit, Clinical Immunology Unit, University of Genova, 16132 Genova, Italy; ^2^Department of Internal Medicine, Respiratory and Allergy Diseases Unit, University of Genova, 16132 Genova, Italy

## Abstract

Allergic diseases are sustained by a T-helper 2 polarization leading to interleukin-4 secretion, IgE-dependent inflammation, and mast cell and eosinophil activation. HLA-G molecules, both in membrane-bound and in soluble forms, play a central role in modulation of immune responses. Elevated levels of soluble HLA-G (sHLA-G) molecules are detected in serum of patients with allergic rhinitis to seasonal and perennial allergens and correlate with allergen-specific IgE levels, clinical severity, drug consumption, and response to allergen-specific immunotherapy. sHLA-G molecules are also found in airway epithelium of patients with allergic asthma and high levels of sHLA-G molecules are detectable in plasma and bronchoalveolar lavage of asthmatic patients correlating with allergen-specific IgE levels. Finally, HLA-G molecules are expressed by T cells, monocytes-macrophages, and Langerhans cells infiltrating the dermis of atopic dermatitis patients. Collectively, although at present it is difficult to completely define the role of HLA-G molecules in allergic diseases, it may be suggested that they are expressed and secreted by immune cells during the allergic reaction in an attempt to suppress allergic inflammation.

## 1. Introduction

The human major histocompatibility complex (HLA) encodes two sets of HLA class I molecules, which have been termed class Ia (or classical) and class Ib (or nonclassical) molecules. The class Ia antigens include the gene products of HLA-A, HLA-B, and HLA-C loci and are characterized by a broad tissue expression and by a high degree of polymorphism [1]. The class Ib antigens include the gene products of HLA-E, HLA-F, and HLA-G loci and are characterized by a restricted tissue distribution and by a limited polymorphism [2]. The encoding genes for classical and nonclassical HLA class I molecules are located on chromosome 6p21 [3]. Since its original description in the 1980s in choriocarcinoma cells and primary cytotrophoblast cells in the placenta [4–6], considerable evidence supports a central role for HLA-G in the suppression of immune responses, long-term immune escape, or tolerance and modulation of inflammation. Allergic diseases are considered as immunoregulatory disorders with decreased tolerance towards allergens [7]. In this review, we discuss the current knowledge and the potential role of HLA-G in allergic diseases.

## 2. HLA-G Structure

 Seven HLA-G isoforms generated by alternative splicing of the primary HLA-G transcript have been characterized. Four of them, HLA-G1, HLA-G2, HLA-G3, and HLA-G4, are bound to the cell surface, while the remaining three, HLA-G5, HLA-G6, and HLA-G7, are detectable in soluble form (sHLA-G). The membrane HLA-G1 molecule, which is derived from the translation of the total HLA-G transcript, and its soluble counterpart HLA-G5 (sHLA-G) have a structure identical to that of classical HLA-A, HLA-B, and HLA-C antigens whereas the other isoforms are smaller lacking one or two domains [8–11]. Truncated isoforms are generated by excision of one or two exons encoding globular domains (G1–G4), while translation of either intron 4 or intron 2 yields sHLA-G5–G7 that lack the transmembrane domain [12]. Furthermore, sHLA-G1 isoform (shed G1 or sHLA-G1) derives from the proteolytic cleavage of the membrane bound HLA-G1. Both HLA-G1 and HLA-G5 have an extracellular structure that is similar to other classical HLA class I molecules: a heavy chain of 3 globular domains that are linked noncovalently to *β*2-microglobulin. By contrast, the other truncated isoforms have a structure similar to HLA class II and do not bind *β*2-microglobulin. Both HLA-G1 and HLA-G5 can homodimerize either in association with *β*2-microglobulin or as free heavy chains. These dimers bind the LIL-RB1/2 (ILT-2/4) receptors for HLA-G with greater affinity as compared with monomeric HLA-G [13, 14]. HLA-G1 and HLA-G5 have been found outside the placenta. HLA-G generally plays immunosuppressive functions and does so by several mechanisms [15]. Membrane-bound HLA-G1 and soluble HLA-G1 and HLA-G5 inhibit uterine and peripheral NK cell activation, CD8+ T-cell mediated cytolysis, and CD4+ T-cell alloproliferative responses. Furthermore, HLA-G may also downregulate alloproliferative responses, induce immune tolerance by promoting the expansion of CD4+ CD25+ FoxP3+ T regulatory (Treg) lymphocytes, and trigger the differentiation of CD4+ T-cells in suppressor cells [15]. While originally described as “highly” restricted in its tissue expression, constitutive expression of HLA-G1 and/or HLA-G5 has been recognized in a number of tissues including thymic medulla, pancreatic islet cells, and peripheral CD14+ mononuclear cells [16, 17]. Both the promoter and the 3′ untranslated region (UTR) of HLA-G are highly polymorphic [5, 6]. Three variations in the 3′UTR, a 14 bp insertion/deletion [18–23], a single nucleotide polymorphism (SNP) substituting guanine at +3142 [24, 25], and a SNP substituting adenine at +3187 [24], can lead to differences in HLA-G expression [12, 25]. However, some polymorphic sites in the 3′UTR of HLA-G interfere with mRNA stability, alternative splicing, and binding of specific microRNAs (miRs) thereby modulating HLA-G mRNA and/or protein expression [26]. Indeed, overexpression of these miRs had also functional activities, since these miRs could directly downregulate HLA-G mRNA and/or HLA-G surface expression. Furthermore, cytokines such as interferon- (IFN-) *γ* and interleukin- (IL-) 10 trigger the expression of HLA-G by peripheral blood mononuclear cells (PBMCs) [27, 28]. IL-10 is a key regulator of immune and inflammatory responses and HLA-G plays an essential role in fetomaternal tolerance by inhibiting lysis by maternal NK cells. IL-10 enhances steady-state levels of HLA-G transcription in cultured trophoblast cells upregulating HLA-G cell surface expression in this cell type. Moreover, IL-10 is able to enhance HLA-G expression and to downregulate classical HLA class I and class II antigens on monocytes, thus regulating NK cells and T lymphocyte responses. These characteristics suggest that IL-10 could be proposed as immunosuppressor agent in the treatment of transplantation rejection and autoimmune diseases [27, 28].

## 3. HLA-G and Allergic Rhinitis

Allergic rhinitis (AR) is sustained by mucosal IgE-dependent inflammation which is promoted, maintained, and amplified by T-helper (Th) 2 cells [29]. The mucosal inflammation is characterized by mast cell and eosinophil activation. Interleukin- (IL-) 4 is a pivotal cytokine that orchestrates allergic inflammation, because it is the most important signal to induce Th2 polarization in allergic patients. IL-4 and IL-13 promote IgE synthesis, upregulate adhesion molecules selective for eosinophil recruitment, and cause increased mucous production and airway hyperreactivity [30–32]. Furthermore, peripheral blood mononuclear cells of AR patients predominantly produce IL-4 [33]. However, a defect in Treg lymphocytes has been demonstrated in allergic patients [7, 34]. Therefore, allergic diseases are considered as immunoregulatory disorders with reduced tolerance towards allergens [7]. These pathophysiologic events promote the production of allergen-specific IgE.

Our group investigated sHLA-A, sHLA-B, sHLA-C, and sHLA-G serum levels in AR patients allergic to* Betula alba*,* Parietaria judaica*, and Graminaceae [35]. sHLA-A, sHLA-B, and sHLA-C serum levels were significantly higher in AR patients as compared to healthy controls (1309 ± 73.3 ng/mL and 1001 ± 145.7 ng/mL, resp., *p* = 0.011). sHLA-G serum levels were also significantly higher in AR patients than in healthy controls (35.86 ± 2.7 ng/mL and 12.79 ± 2.4 ng/mL, resp., *p* < 0.0001). Moreover, we found a moderate but significant correlation between sHLA-G and sHLA-A, sHLA-B, and sHLA-C levels in AR patients (*r* = 0.37). Serum sHLA-G levels were also evaluated in patients with AR due to perennial allergens including house dust mite and cat and dog dandruff [36, 37]. Clinical severity was evaluated with a validated visual analogue scale (VAS) for quantifying the perception of nasal symptoms intensity and drug consumption at the end of the pollen season [38]. The serum levels of sHLA-G molecules resulted as significantly higher in patients with perennial AR than in healthy controls (*p* < 0.0001). Notably, there was a strong correlation between sHLA-G serum levels, VAS score (*r* = 0.850, *p* < 0.001), and drug use (*r* = 0.793, *p* < 0.001). Notably, a significant even though weak correlation between serum sHLA-A, sHLA-B, and sHLA-C levels and VAS was also observed (*r* = 0.309, *p* = 0.016) but not between serum sHLA-A, sHLA-B, and sHLA-C levels and drug consumption [37]. A further study showed that serum sHLA-G levels were significantly associated with allergen-specific IgE levels both in allergic rhinitis (*r* = 0.468 and *p* = 0.003) and in allergic asthma (*r* = 0.479 and *p* = 0.006) patients [39]. Finally, serum sHLA-G levels were higher in patients with seasonal allergy than in those with perennial allergy (*p* = 0.0194) [40]. Data from another group confirmed that serum sHLA-G levels are significantly higher also in children with allergic diseases [41].

AR management includes patient education, allergen avoidance, drug treatment, and, when appropriate, allergen-specific immunotherapy [42]. The aim of allergen-specific sublingual immunotherapy (SLIT) is to achieve clinical tolerance to the causal allergen through oral administration of high-dose allergens by shifting Th2 immune response, mainly mediated by IL-4, to Th1 response, mainly mediated by interferon- (IFN-) *γ*. We evaluated sHLA-G and sHLA-A, sHLA-B, and sHLA-C serum levels before and 3 months after the end of SLIT and correlated their values with IFN-*γ* production by peripheral blood mononuclear cells [43, 44]. sHLA-G levels decreased from 35.86 ± 2.7 ng/mL to 11.36 ± 1.6 ng/mL (*p* < 0.0001) and sHLA-A, sHLA-B, and sHLA-C levels decreased from 1309 ± 73.3 ng/mL to 695.3 ± 33.2 ng/mL (*p* < 0.0001). Notably, IFN-*γ* production increased from 44 ± 68 spots before SLIT to 181 ± 89 spots after SLIT (*p* < 0.0001) and significantly correlated with both sHLA-G (*r* = −0.39, *p* = 0.023) and sHLA-A, sHLA-B, and sHLA-C (*r* = −0.38, *p* = 0.029) changes. Furthermore, the percentage changes of sHLA-G and sHLA-A, sHLA-B, and sHLA-C levels were significantly correlated among themselves (*r* = 0.84) and with VAS score (*r* = 0.63 and *p* = 0.60, resp.).

## 4. HLA-G and Asthma

Persistent airway inflammation, structural remodelling, and bronchial hyperresponsiveness in lower airways are hallmarks of allergic asthma [45, 46]. Allergen-driven activation of Th2 CD4+ T-lymphocytes releasing IL-4, IL-5, and IL-13 perpetuate the inflammation via recruitment of other lymphocytes, eosinophils, and mononuclear cells [47–49]. Genetic factors play a central role in asthma pathogenesis. Indeed, over 100 genes have been implicated in asthma susceptibility [50]. One of such genes may be HLA-G. In fact, HLA-G genetic polymorphisms confer susceptibility to airway hyperresponsiveness and asthma development [3]. The G/G genotype at SNP-964 in the promoter region 4 is associated with asthma in the offspring of mothers with asthma or bronchial hyperresponsiveness (BHR), while the A/A genotype was associated with asthma in the offspring of asthma- and BHR-free mothers [37]. Tan et al. [51] demonstrated that the SNP-964G/A tagged two major promoter haplotype clades with evidence of longstanding balancing selection amongst African Americans, European Americans, and Han Chinese individuals. There is a strong linkage disequilibrium between SNPs in the HLA-G gene. The +3142 C/G SNP, which is located in the 3′UTR of the HLA-G gene, affects the targeting of miR-148a, miR-148b, and miR-152 and interacts with mother's asthma status to determine risk of asthma in the child [24]. Notably, the +3142 (rs10633320) G/G SNP resulted as protective against asthma among offspring of asthmatic mothers whereas the C allele was associated with asthma risk in the offspring of mothers without asthma. Statins upregulate miR-148b and miR-152 and, thus, affect HLA-G expression. It has been found that subjects carrying at least one copy of the G minor allele of the rs10633320 presented a decreased frequency of asthma-related exacerbations (emergency department visit, hospitalizations, or oral corticosteroid use). Moreover, there was no difference in exacerbation frequency between G/G and G/C genotypes [52]. A genome-wide association study (GWAS) confirmed in 6819 participants from the Framingham Heart Study the association of previously described genetic variants in FCERIA, STAT6, and IL-13 and identified potential susceptibility loci in the HLA-A, HLA-G, and HLA-DQA2 gene regions as risk factors for IgE dysregulation and atopy [53]. KIR2DL4 (CD158d) is a member of the killer cell immunoglobulin-like receptor family that is mainly expressed on natural killer (NK) cells and its ligand has been reported to be HLA-G [54–58]. Furthermore, NK-cell derived IFN-*γ* secretion has been reported to be critical for the generation of tolerogenic dendritic cell (DC) in the placenta [59]. It might be predicted that individuals with the functionally defective 9A allele of KIR2DL4 would not be able to secrete IFN-*γ* and might therefore be prone to producing Th2-biased immune responses and fewer tolerogenic DC. KIR2DL4 genotypes were analyzed in 2 cohorts of children at high risk for atopic disease [60]. However, there was neither significant relationship between KIR2DL4 genotype and the prevalence of atopy, as assessed by allergen skin prick testing in either cohort at any age, nor was there any significant relationship between KIR2DL4 genotype and the prevalence of wheeze, bronchial hyperreactivity, and asthma. A role for HLA-G in asthma pathogenesis was further suggested by the demonstration of the expression of the sHLA-G5 isoform in the airway epithelium and of increased circulating plasma levels of sHLA-G in children with atopic asthma [61]. Because airway inflammation in asthma involves a Th-2 skewing of lymphocytes similar to pregnancy, HLA-G is an attractive candidate molecule for promoting the immune profile characteristic of asthma. Localization of HLA-G in airway epithelium suggests that it could contribute to immune dysregulation and airway inflammation in chronic asthma. Tahan and Patiroglu [61] measured sHLA-G levels in bronchoalveolar lavage (BAL) of patients with mild persistent asthma and found increased levels as compared to controls (*p* = 0.01). Notably, there was no significant difference in sHLA-G BAL levels in Caucasian individuals as compared to African-American individuals. Furthermore, sHLA-G was present in the epithelium of endobronchial biopsies from 9 of 11 patients with asthma. These findings supported and confirmed a role for sHLA-G in asthma pathogenesis. Zheng et al. [62] confirmed that plasma sHLA-G levels were significantly higher in atopic asthmatic children than in healthy controls (*p* < 0.001). However, no significant association was observed between plasma sHLA-G, total IgE, and allergen-specific IgE levels. Moreover, sHLA-G levels were not significantly related to HLA-G 14 bp insertion/deletion polymorphism both in asthmatic children and in controls. On the contrary, we found a significant association between sHLA-G levels and allergen-specific IgE levels both in AR (*r* = 0.468 and *p* = 0.003) and in asthmatic (*r* = 0.479 and *p* = 0.006) patients [36]. Mapp et al. [63] demonstrated that baseline levels of IL-10 secretion by PBMC in patients with isocyanate-induced asthma and asymptomatic-exposed individuals are higher than those in nonoccupational allergic asthma and in healthy controls (*p* < 0.0001). Spontaneous production of sHLA-G by PBMCs resulted as significantly higher in patients with isocyanate-induced asthma than in the other groups (*p* < 0.005).

## 5. HLA-G, Allergy and Pregnancy

Rizzo et al. [64] evaluated the potential role of pregnancy and labor on plasma sHLA-G and IL-10 levels in women with AR or asthma and in healthy pregnant women. Plasma samples were obtained during the 3rd trimester of pregnancy, at delivery, and at a nonpregnant state 2 years postpartum. The plasma levels of sHLA-G1 isoform and IL-10 resulted as significantly increased during labor in comparison with the levels detected during the 3rd trimester and 2 years after delivery (*p* < 0.0001). However, allergic women had lower plasma sHLA-G levels than nonallergic women during the 3rd trimester of pregnancy and at delivery (*p* < 0.01 and *p* < 0.05, resp.). Interestingly, no significant differences were found in samples obtained 2 years after pregnancy. Thymus-and-activation-regulated chemokine (TARC or CCL17) is one of the Th2-inducible chemokines produced by the thymus [65] and by trophoblasts and endometrial gland cells during pregnancy [65]. CCL17 is related to both allergy and pregnancy. Miyahara et al. [66] enrolled 70 paired full-term and normal-vaginal-delivery newborns and their mothers and reported that serum levels of CCL17 were higher in mothers with atopic dermatitis (AD) than in those without AD (*p* < 0.001). High umbilical cord serum levels of CCL17 were associated with infantile AD development (*p* < 0.001). Serum levels of CCL17 (*r*s = 0.340, *p* < 0.001) and sHLA-G (*r*s = 0.600, *p* < 0.001) showed high correlations between umbilical cord and maternal blood.

## 6. HLA-G and Atopic Dermatitis

Atopic dermatitis (AD) is a chronic disease usually beginning in childhood. AD is characterized by increased production of IL-4, IL-13, and IgE [67]. In AD biopsies, HLA-G positive cells were always found in the papillary and, less frequently, in the reticular dermis [64]. HLA-G was expressed mainly by infiltrating T cells but also, to a lesser extent and less frequently, by monocytes-macrophages or Langerhans cells [68].

## 7. What Is the Role of HLA-G in the Pathogenesis of Allergic Diseases?

HLA-G molecules have a complex immune regulatory role in transplantation, cancer, viral infections, chronic inflammatory diseases, and pregnancy [8, 9, 69–72]. In general, HLA-G is a tolerance-inducing molecule but it is also a stimulus for Th2 responses and Treg cells activation [73]. Allergic diseases are driven by a Th2-polarized inflammation [74] and allergic patients display a defect in Treg cells which may be restored by specific immunotherapy [34]. At present, it is difficult to completely clarify the role of HLA-G in allergic diseases. It may be suggested that they are expressed and secreted by immune cells during the allergic reaction and may represent a reactive attempt to suppress allergic inflammation. This hypothesis is supported by their increase during immunotherapy and is in keeping with the finding that antigen presenting cells and monocytes expressing HLA-G molecules are able to create a tolerogenic milieu enriched in IL-10 which, in turn, promotes Treg cells activity [75, 76]. HLA-G genetic polymorphisms confer susceptibility to airway hyperresponsiveness and asthma development. In particular, G/G genotype at SNP-964G/A in the promoter region is associated with asthma; the +3142 C/G SNP increases the risk of asthma in the child. By contrast, the +3142 (rs10633320) G/G SNP resulted as protective against asthma development. In conclusion, it could be postulated that increased HLA-G levels could be either compensatory or pathogenetic through mechanisms not yet completely known. Accordingly, it has been recently proposed that HLA-G should be no longer qualified as a “shield” to protect tissues and cells from immune destruction but, rather, as an “immune checkpoint” molecule [77].
